# 27-Hydroxycholesterol Binds GPER and Induces Progression of Estrogen Receptor-Negative Breast Cancer

**DOI:** 10.3390/cancers14061521

**Published:** 2022-03-16

**Authors:** Paola Avena, Ivan Casaburi, Lucia Zavaglia, Marta C. Nocito, Davide La Padula, Vittoria Rago, Jing Dong, Peter Thomas, Chieko Mineo, Rosa Sirianni, Philip W. Shaul

**Affiliations:** 1 Department of Pharmacy, Health and Nutritional Sciences, University of Calabria, Arcavacata di Rende, 87036 Cosenza, Italy; paola.avena@unical.it (P.A.); ivan.casaburi@unical.it (I.C.); luciazavaglia@hotmail.it (L.Z.); nocitomarta90@tiscali.it (M.C.N.); davidelapadula@live.it (D.L.P.); vittoria.rago@unical.it (V.R.); 2 Marine Science Institute, University of Texas at Austin, Port Aransas, TX 78373, USA; jingdong@mail.utexas.edu (J.D.); peter.thomas@utexas.edu (P.T.); 3 Center for Pulmonary and Vascular Biology, Department of Pediatrics, University of Texas Southwestern Medical Center, Dallas, TX 75390, USA; chieko.mineo@utsouthwestern.edu

**Keywords:** GPER, breast cancer, 27-hydroxycholesterol, angiogenesis

## Abstract

**Simple Summary:**

Breast cancer is the most common cancer in women, and there is a known link between high cholesterol levels and breast cancer. However, how cholesterol impacts breast cancer is poorly understood, particularly in the case of an aggressive form of cancer known as estrogen receptor negative breast cancer. Using cells in culture and models of breast tumors in mice, we have determined that an abundant metabolite of cholesterol known as 27-hydroxycholesterol stimulates estrogen receptor negative breast cancer growth. We have also determined how 27-hydroxycholesterol stimulates the growth, identifying a new mechanism of action of 27-hydroxycholesterol. These new findings may explain the link between high cholesterol and estrogen receptor negative breast cancer, and they may lead to the development of new therapies for a type of breast cancer that presently lacks specific treatments.

**Abstract:**

Cholesterol affects the proliferation of breast cancer (BC) and in particular of estrogen receptor-negative (ER−) BC. Cholesterol is converted to 27-hydroxycholesterol (27HC), which promotes the growth of ER+ BC. Potentially, 27HC can be involved in cholesterol-dependent ER− BC proliferation. Stable MDA-MB-231 silenced clones for CYP7B1 (27HC metabolizing enzyme) show an increased basal proliferation rate, which is not observed in the presence of lipoprotein-deprived serum. Furthermore, the treatment of SKBR3, MDA-MB-231 and MDA-MB-468 with 27HC increased cell proliferation that was prevented by G15, a selective G Protein-Coupled Estrogen Receptor (GPER) inhibitor, suggested this receptor to be a potential 27HC target. Binding experiments demonstrate that 27HC is a new ligand for GPER. We show that ERK1/2 and NFκB are part of the 27HC/GPER pathway. The stable silencing of GPER prevents NFκB activation and reduces basal and 27HC-dependent tumor growth. Additionally, conditioned medium from ER− BC cells treated with 27HC promotes tube formation, which does not occur with CM from GPER silenced cells. Collectively, these data demonstrate that cholesterol conversion into 27HC promotes ER− BC growth and progression, and the expression of GPER is required for its effects.

## 1. Introduction

Breast cancer (BC) is the most common cancer among women, and despite improvements in early diagnosis and treatment, is the second-highest cause of cancer death. Classification of BC has commonly been related to the expression of estrogen receptor (ER), progesterone receptor (PR), and human epidermal growth factor receptor 2 (HER2). Recently, gene expression profiling by DNA microarray analysis has led to the classification of breast tumors into five or six subtypes [1,2,3]. These classifications can guide treatment and predict responses to therapy. Tumors that are ER− and do not express the PR or HER2 (triple negative, TNBC) are the most difficult to treat and have the worst prognosis [2,3]. Lacking a well-defined receptor and pathway signature, chemotherapy remains the treatment of choice, but the recurrence rate is still high. Among the potential factors associated with breast cancer, cholesterol has recently received considerable attention. Epidemiologic studies have provided inconclusive results, indicating that there may be a relationship between abnormal plasma cholesterol levels and breast cancer risk. However, more compelling evidence has been obtained in laboratory studies, and these studies indicate that cholesterol is able to regulate proliferation, migration, and signaling pathways in breast cancer. Many groups were able to show that lipoproteins stimulated the growth of breast cancer cells in vitro [4,5,6,7]. The pro-proliferative effect of LDL appears to be dependent on the ER status since only ER− breast cancer cell lines display increased proliferation in the presence of LDL [6].

Cholesterol can be metabolized to oxysterols, which control the feedback regulation of cholesterol biosynthesis. Among them, 27-hydroxycholesterol (27HC) has been associated with the growth and metastases of several cancers [8]. The oxysterol, 27HC, exerts its effects by binding to liver X receptors (LXR), alpha and beta [9,10] and estrogen receptor-α (ERα) [11,12]. Since its identification as a SERM, 27HC has been implicated in several diseases, including atherosclerosis, osteoporosis, cancer, and Alzheimer’s disease [13]. In the context of ER+ breast tumors, the binding of 27HC to ERα increases cancer growth [14,15], while the activation of LXRα is involved in pro-metastatic effects [15]. In the context of ER− BC, it has been shown that monocytes present at the primary site are responsible for the production of 27HC [16], which can be utilized by ER− BC cells to increase migratory ability [17]. Importantly, CYP27A1, the enzyme responsible for 27HC synthesis, is heterogeneously expressed among primary tumors, with a high expression significantly associated with a high tumor grade, ER negativity, and basal-like subtypes [18]. On the contrary, tumor cells from patients with TNBC display a low immunoreactivity for CYP7B1, the enzyme responsible for the degradation of 27HC [19]. Furthermore, in ER− BC, 27HC induces reactive oxygen species (ROS) production and Stat-3 activation to promote VEGF production and angiogenesis [20], a necessary event in the metastatic processes. The two known receptors binding 27HC are unlikely to mediate its effects since, by definition, these tumors are ER negative and the activation of LXRα represses ER− BC proliferation [21]. Despite the role of 27HC in ER− BC, the receptor mediating its effects in this context is still unknown.

GPR30, a G-protein-coupled receptor encoded by the GPER (G-protein estrogen receptor) gene, has been linked to estrogen effects in several physiological and pathological settings [22]. Different from nuclear ER knockout mice, GPER KO mice did not manifest an infertile phenotype. However, GPER was implicated in obesity, insulin resistance, cardiovascular dysfunction, and breast cancer progression [22]. GPER is prevalent in TNBC and presumed to be involved in the growth of this tumor, becoming a potential candidate as a therapeutic target [23,24,25,26,27,28]. In two TNBC cell lines, MDA-MB-435 and HCC1806, GPER activation by its agonists, estradiol, and tamoxifen increased proliferation, and GPER silencing completely prevented this effect [24]. Additionally, GPER was strongly expressed in the TNBC cell lines, MDA-MB-468 and MDA-MB-436, and its agonists led to the rapid activation of the ERK1/2 pathway, which is responsible for increased cell growth, survival, migration, and invasion through upregulating the expression of genes associated with cell cycle, anti-apoptotic and proliferative mechanisms [26]. In vitro experimental results were supported by clinical data from TNBC patients, indicating a strong association between positivity for GPER and pERK1/2 and large tumor size and advanced stage [24]. Additionally, in ER negative BC cells, GPER increases VEGF production to support angiogenesis [29].

Altogether, these observations highlight an overlap between GPER- and 27HC-dependent effects. Therefore, in this study, we asked if 27HC could bind and activate GPER to increase the metastatic potential of ER− BC.

## 2. Materials and Methods

### 2.1. Cell Culture and Clones Selection

The human breast cancer cell lines, SKBR3 and MDA-MB-231, were obtained from the American Type Culture Collection (ATCC, Manassas, VA, USA). MDA-MB-468 cells were a gift from Prof. Richard Pestell (Department of Cancer Biology, Thomas Jefferson University, Philadelphia, PA, USA). SKBR3 human breast cancer cells were maintained in phenol red-free RPMI 1640 containing 10% fetal bovine serum (FBS) and antibiotics (Pen/Strep), and the TNBC cell lines, MDA-MB-231 and MDA-MB-468, were maintained in DMEM/F- 12 containing 10% FBS and 1 mg/mL penicillin-streptomycin. Cells were maintained in phenol red-free media containing 5% FBS-DCC (fetal bovine serum, dextran-coated charcoal-treated) for three days before being plated for specific experiments using phenol red-free media with 0.5% FBS-DCC. During experimental treatments, the cells were grown in phenol red-free media containing 5% FBS-DCC. Additionally, specific experiments were performed on cells grown in a medium containing lipoprotein-free serum (LPF-FBS) (Sigma-Aldrich, Inc. St. Louis, MO, USA).

Stable MDA-MB-231 and MDA-MB-468 cell lines were created, harboring control shRNA or shRNA-targeting GPER. These cell lines were generated by transfecting MDA-MB-231 cells with short hairpin RNA (shRNA) plasmids (pGFP-V-RS HuSH vector; Ori- gene, Rockville, MD, USA). Stable clones were selected using 10 μg/mL puromycin and maintained in growth medium containing 1µg/mL of puromycin (Millipore Sigma, St. Louis, MO, USA). GPER mRNA levels were determined by real-time PCR using the following primers: Forward, 5′-CGCTCTTCCTGCAGGTCAA-3′, and reverse, 5′-ATGTAGCGGTCGAAGCTCATC-3′; they were normalized to 18S ribosomal RNA. GPER protein levels in each clone were determined by Western blotting using an anti-GPER antibody (MBL International Corporation, Woburn, MA, USA).

Stable MDA-MB-231 cell lines were created, harboring control shRNA or shRNA-targeting CYP27A1 or CYP7B1. These cell lines were generated by infecting MDA-MB-231 cells with lentiviral particles containing three to five expression constructs, each encoding target-specific 19–25 nt (plus hairpin) shRNA designed to knock down gene expression (Santa Cruz Biotechnology Inc., Dallas, TX, USA). Stable clones were selected using 10 μg/mL puromycin and maintained in a growth medium containing 1μg/mL of puromycin (Sigma-Aldrich, Inc. St. Louis, MO, USA). CYP27A1 and CYP7B1mRNA levels were determined by real-time PCR using the following primers: CYP27A1- Forward, 5′-TGCGCCAGGCTCTGAACCAG-3′, and Reverse, 5′-TCCACTTGGGGAGGAAGGTG-3′; CYP7B1 Forward, 5′-GGCCCTCTGCTTGCTTGTC-3′, and Reverse, 5′-GAAGCCAACCTTTTATCAATGGA-3′; they were normalized to 18S ribosomal RNA.

The human embryonic kidney HEK293 cell line was obtained from ATCC. For use in GPER competitive binding assays, human HEK293 cells were stably transfected with GPER [30], cells expressing GPER were selectively maintained with 500 μg/mL geneticin, and the cells were cultured in DMEM/Ham’s F-12 medium without phenol red supplemented with 5% fetal bovine serum (FBS) and 100 μg/mL of gentamicin in 150 mm diameter plates. The medium was replaced every 1–2 days, and the cells reached 80% confluence after 3 days in culture, at which time the medium was replaced with medium-lacking FBS, and the cells were cultured for another day before binding experiments were performed.

### 2.2. Proliferation and Viability Quantitation

SKBR3, MDA-MD-231, and MDA-MD-468 cell proliferation was assessed by quantifying [^3^H]thymidine incorporation (Perkin-Elmer Life Sciences, Boston, MA, USA) as previously described [14]. Cell viability was measured using 3-[4,5-dimethylthiazol-2-yl]-2,5-diphenyltetrazolium bromide (MTT) assay as previously described. Fresh MTT (Sigma-Aldrich, Inc. St. Louis, MO, USA), resuspended in PBS, was then added to each well (final concentration 0.33 mg/mL). After 2 h incubation, cells were lysed with 200 µL of DMSO, and optical density was measured at 570 nm in a multi-plate reader.

### 2.3. Western Blot Analysis

Following cell treatment, total protein extraction was performed using RIPA buffer as previously described [31]. Proteins were separated by 10% SDS-PAGE and immunoblotted overnight with specific antibodies against pERK1/2 (Cell Signaling Technology, Danvers, MA, USA) or total ERK1/2 (Cell Signaling Technology). In studies of NF-kB activation, following treatment, cytoplasmic and nuclear extracts were prepared from cultured cells [32], and immunoblotting was performed using antibodies against NF-κB p65 (Abcam, Cambridge, UK), glyceraldehyde 3-phosphate dehydrogenase (GAPDH), or Lamin B (Santa Cruz Biotechnology, Dallas, TX, USA). Membranes were incubated with horseradish peroxidase (HRP)-conjugated secondary antibodies (Santa Cruz Biotechnology, Dallas, TX, USA), and immunoreactive bands were visualized with the ECL Western blotting detection system (Santa Cruz Biotechnology, Dallas, TX, USA).

### 2.4. RNA Extraction, Reverse Transcription and Real-Time PCR

Following total RNA extraction, 1 µg of total RNA was reverse transcribed and then used for PCR reactions, performed in the QuantStudio 3 Real-Time PCR System (Applied Biosystem, Waltham, MA, USA). Final results were expressed as n-fold differences in gene expression relative to 18S and calibrator and calculated using the ΔΔCt method as previously shown [31]. Primers sequence are indicated in Table 1.

### 2.5. GPER Competitive Binding Assay

[^3^H]E2 binding to plasma membranes of HEK293 cells transfected with GPER was evaluated as previously reported [30]. Specific E2 binding was calculated from the binding of 4 nM [2,4,6,7-^3^H]E2 ([^3^H]E2, ~89 Ci/mmol) alone (total binding) and in the presence of 100-fold excess (400 nM) nonradioactive E2 (nonspecific binding). Competitive binding assays were run in triplicate with 4 nM [^3^H]E2, and competitor was added over a concentration range of 1 nM to 10 μM (dissolved in 1 μL ethanol). After a 30 min incubation at 4 °C with the plasma membrane fractions, the reaction was stopped by filtration (Whatman GF/B filters), filters were washed, and bound radioactivity was measured by scintillation counting. The displacement of [^3^H]E2 binding by the competitor was expressed as a percentage of the maximum specific binding of E2. The competition curves and IC50 values (competitor concentrations that cause 50% displacement of [^3^H]-E2) were calculated from the mean values from three separate competitive binding assays.

### 2.6. Mammosphere Formation Assay

A single-cell suspension was prepared using enzymatic (Trypsin-EDTA, Millipore Sigma, #T3924) and manual disaggregation (25-gauge needle). Cells were plated in 6-well tissue culture plates covered with poly-2-hydroxyethyl-methacrylate (poly-HEMA, Millipore Sigma, #P3932) to prevent cell attachment, at a density of 0.5 × 10^3^ cells/mL in mammosphere medium (DMEM-F12/B27/EGF (20 ng/mL)/Pen-Strep/Glu) [33]. Cells were grown for 5 days and maintained at 37 °C in a humidified atmosphere containing 5% CO_2_ in the absence or presence of treatments. After 5 days of culture, spheres > 50 μm were counted using an eyepiece graticule. The percentage of plated cells that generated spheres was calculated to yield the percentage mammosphere formation, and values are expressed relative to those for basal conditions equaling one (1 = 100% MFE, mammosphere forming efficiency).

### 2.7. Mouse Studies

#### 2.7.1. Ethics Statement

All protocols were approved by the University of Texas Southwestern Medical Center Institutional Animal Care and Use Committee (APN#2013-0128). All institutional guidelines for the care and use of laboratory animals were followed.

#### 2.7.2. Orthotopic Breast Tumor Xenograft Model

Non-manipulated MDA-MB-231, or stable MDA-MB-231 cell lines harboring control shRNA or shRNA-targeting GPER (1.5 × 10^6^ cells) were implanted unilaterally into the mammary fat pad of 6-week-old female SCID mice (Harlen Envigo). Tumor volume was then measured intermittently with calipers. In studies of 27HC, when non-manipulated MDA-MB-231 cell tumors reached an average volume of 100 mm^3^, treatment was initiated with vehicle or 100 µg 27HC injected subcutaneously every other day [14]. At 28 d, the tumors were harvested and weighed.

### 2.8. Histopathological Analysis and Staining

At tumor harvest, formalin-fixed, paraffin-embedded tissues were sectioned at 5 μm, mounted on slides precoated with polylysine, and stained with hematoxylin and eosin. Sections were deparaffinized and dehydrated. Slides were permeabilized with 0.2% Triton X-100, followed by blocking with serum (30 min, room temperature), and incubated with anti- Ki67 (Abcam), Endomucin (BioRad, Hercules, CA, USA), CD31 (Biolegend, San Diego, CA, USA), Vimentin (Dako, Copenhagen, Danmark) antibody, and diaminobenzidine-conjugated secondary antibody (1:200, 30 min, room temperature). Secondary antibody alone was used as a negative control. Slides were observed and photographed with an OLYMPUS BX51 microscope.

### 2.9. Conditioned Medium

Breast cancer cells were seeded at 250,000 cells/well in a 6-well plate in a medium containing 5% FBS-DCC for 3 days, cells were washed and incubated for additional 12 h with medium without serum, before treatment for 24 h with 27HC resuspended in DMSO in medium containing 1% FBS-DCC. Thereafter, the supernatants were collected, centrifuged at 3500 rpm for 5 min to remove cell debris, and used as a conditioned medium for EA.hy926. Stably silenced breast cancer clones were seeded at 250,000 cells/well in a 6-well plate in a medium containing 5% FBS-DCC for 3 days, supernatants were collected, centrifuged at 3500 rpm for 5 min to remove cell debris, and used as conditioned medium for EA.hy926.

### 2.10. Tube Formation Assay

The day before the experiment, confluent EA.hy926 were starved overnight at 37 °C in serum-free medium. Growth factor-reduced Matrigel^®^ (R&D Systems Inc. Minneapolis, MN, USA) was thawed overnight at 4 °C, plated on the bottom of prechilled 96-well plates and left at 37 °C for 1 h for gelification. Starved EA.hy926 were collected by enzymatic detachment (0.25% trypsin-EDTA solution, Life Technologies, Carlsbad, CA, USA), counted and resuspended in conditioned medium from breast cancer cells. Then, 10,000 cells/well were seeded on Matrigel and incubated at 37 °C. Tube formation was observed starting from 4 h after cell seeding and quantified using the software NIH ImageJ (National Institutes of Health (NIH), Bethesda, MD, USA).

### 2.11. Statistical Analysis

All cell culture and xenograft experiments were performed on at least three separate occasions. Data are expressed as mean ± standard error of the mean (SEM), and statistical analysis was performed using GraphPad Prism 5.0 software (GraphPad Software, version 7.0 La Jolla, CA, USA). Comparisons between two groups were conducted using Student’s *t*-test, and comparisons between 3 or more groups were conducted by analysis of variance (ANOVA) with Newman–Keuls Multiple Comparison post hoc testing. Xenografts growth curve analysis was conducted using Two-way ANOVA followed by Bonferroni post hoc testing. Significance was defined as *p* < 0.05.

## 3. Results

### 3.1. TNBC Cells Convert Cholesterol to 27HC to Increase Tumor Growth

**Figure 1:**
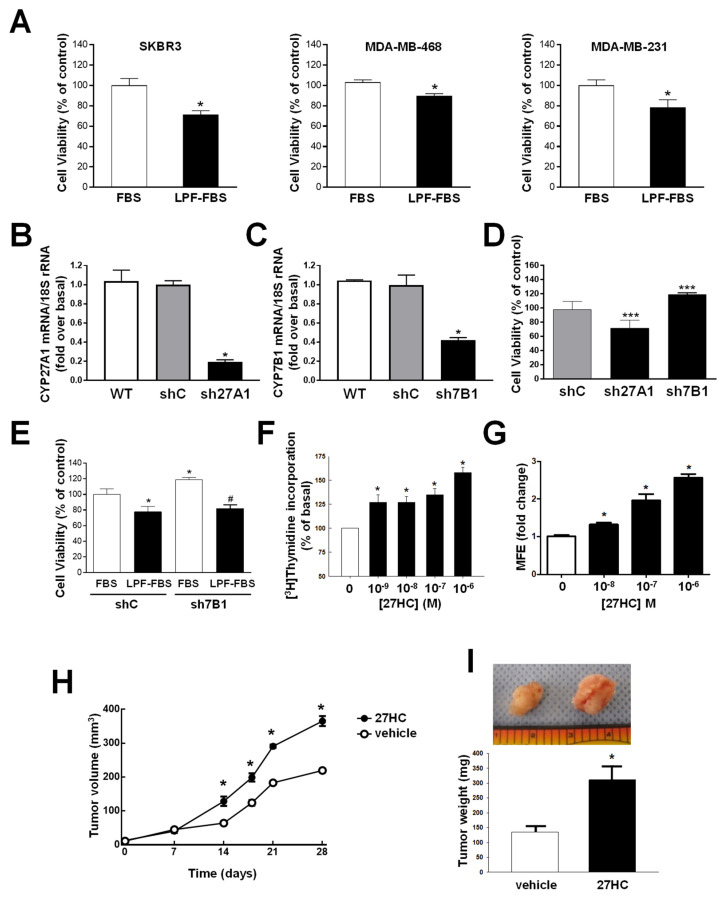
Cholesterol metabolism to 27HC increases ER− BC growth. (**A**) SKBR3, MDA-MB-468 and MDA-MB-231 were grown for 96 h in the presence of 5% FBS or lipoprotein-free FBS (LPF-FBS) (SIGMA), and cell viability was evaluated by MTT assay. * *p* < 0.05 vs. FBS. (**B**,**C**). MDA-MB-231 were stably silenced by CYP27A1 (sh27A1) (**B**) and CYP7B1 (sh7B1) (**C**) using gene-specific shRNA. (**D**) Cell viability of sh27A1 and sh7B1 cells was evaluated by MTT assay in comparison to shcontrol (shC) cells at 96 h. *** *p* < 0.001 vs. shC. (**E**) ShC and sh7B1 cells were grown for 96 h in the presence of 5% FBS or 5% LPF-FBS. * *p* < 0.05 vs. shC-FBS; # *p* < 0.05 vs. sh7B1-FBS. (**F**) Proliferation was evaluated by ^3^H-thymidine incorporation in MDA-MB-231 cells treated for 48 h with increasing concentrations of 27HC, * *p* < 0.05 vs. 0 µM 27HC. (**G**) MDA-MB-231 cells were cultured under non-adherent conditions and grown in the presence of vehicle (0 M) or 27HC (10^−8^, 10^−7^, 10^−6^ M) for 5 days, and spheres > 50 µm were quantified. Findings are expressed relative to mammosphere formation efficiency (MFE) under basal conditions (0 M). Values are mean±SEM, n = 9, * *p* < 0.05 vs. vehicle. (**H**,**I**). Xenografts were initiated using MDA-MB-231 cells, and the mice were administered vehicle versus 27HC for 28 days. (**H**) Growth curves of tumors measured by caliper. * *p* < 0.05 vs. 0 µM 27HC. (**I**) Representative tumors and final tumor weights. Values are mean ± SEM, n = 7, * *p* = 0.009 vs. vehicle.

LDL-cholesterol increases the growth of TNBC [4,34]. We evaluated the growth of BC cells in medium containing regular FBS or lipoprotein-free FBS (LPF-FBS). After 96 h in culture, a significant decrease in the proliferation rate of the three tested ER− cell lines was observed (Figure 1A).

To define the role of 27HC in the cholesterol-dependent effect on TNBC growth, the CYP27A1 gene, encoding for the 27HC synthesizing enzyme (shCYP27A1, sh27A1), and the CYP7B1 gene, encoding for 27HC metabolizing enzyme (shCYP7B1, sh7B1), were stably silenced in MDA-MD231 cells (Figure 1B,C). These two cell lines have proliferation rates that differ from control cells (shcontrol, shC). ShCYP27A1 cells grow slower, with a 30% decrease in the proliferation rate, while shCYP7B1 cells grow faster and manifest a 20% increase compared to shC cells (Figure 1D). Importantly, the increased proliferation rate observed for shCYP7B1 cells requires cholesterol (Figure 1E). Cyclin D1 and E expression is increased in shCYP7B1 versus shC cells when cholesterol is available (FBS) (Figure S1A–C). This difference cannot be appreciated in the absence of cholesterol (LPF-FBS) (Figure S1D–F). Collectively, these data demonstrate that, in TNBC, cholesterol is converted to 27HC and increases tumor growth.

Treatment with exogenous 27HC increased the proliferation of the three cell lines (Figure 1F–H). Additionally, 27HC favored breast cancer growth as mammospheres (Figure 1G and Figure S2A,B). The in vitro data were confirmed in vivo and MDA-MB-231 were grafted in the mammary fat pad of mice treated for 4 weeks with either a vehicle or 27HC, which was able to increase both tumor volume and weight (Figure 1H,I).

Similar in vivo data were obtained by treating mice grafted with MDA-MB-468 with 27HC (Figure S2C,D).

### 3.2. 27HC Binds to GPR30 and Mediates ERK1/2 and NFκB Activation to Increase Tumor Proliferation

**Figure 2:**
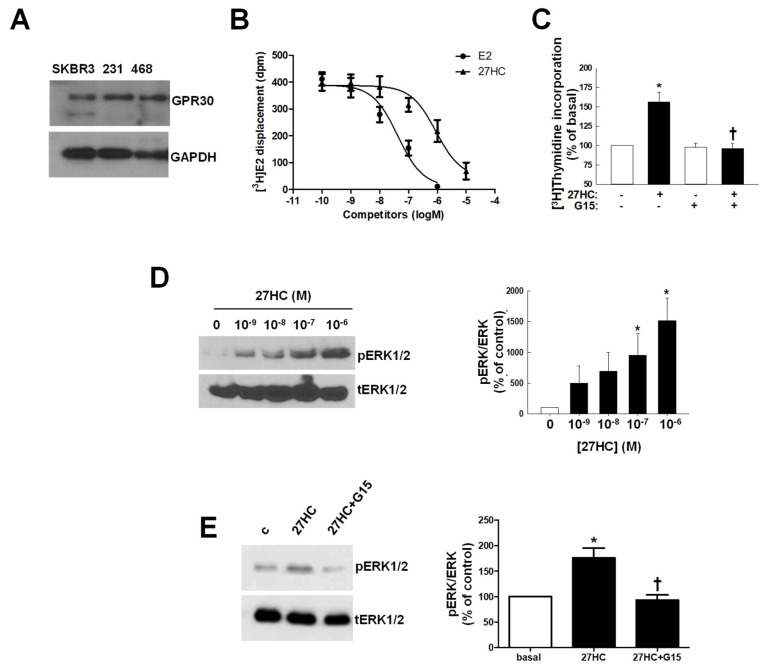
27HC binds to GPR30 and activates ERK1/2. (**A**). GPR30 expression was evaluated in whole-cell lysates from SKBR3, MDA-MB-231 (231) and MDA-MB-468 (468) cells. (**B**) Estradiol (E2) and 27HC competitive binding to GPR30 was evaluated using plasma membranes isolated from GPR30-expressing HEK293 cells. [^3^H]E2 displacement is shown in the presence of increasing concentrations of E2 or 27HC. Values are mean ± SEM, n = 3. (**C**). Cells were treated for 48 h with 27HC 10^−6^ M with or without G15 (10^−6^ M), * *p* < 0.05 vs. basal (untreated cells); † *p* < 0.05 vs. 27HC. (**D**,**E**) Western blot analysis was performed to detect posphorylated ERK1/2 (pERK1/2) or total ERK1/2 (tERK1/2) on whole-cell lysates from MDA-MB-231 cells treated for 15 min with 0 to 10^−6^ M 27HC (**D**) or with 27HC 10^−6^ M and G15 10^−6^ M alone or in combination (**E**). Left panels display representative Western blots, and right panels provide summary data. Values are mean ± SEM, n = 3.

The observed 27HC effects are unlikely to be related to the known 27HC receptors: LXR and ERα. Our models are ER− and previous reports indicated that LXRα activation decreases the proliferation of MDA-MB-231 and SKBR3 [21] and MDA-MB-468 [35]. Accordingly, LXR synthetic agonist, T1317, reduced the proliferation of all three cell lines. We considered the possibility of 27HC binding to GPER since the activation of this receptor has been reported to increase the growth of ER− BC cells [36]. We confirmed that all three cell lines used in the study express the GPER protein (GPR30) (Figure 2A). Binding studies demonstrated that 27HC competes for [^3^H]E2 binding to GPER with an IC50 of 0.50–0.80 µM (Figure 2B). The addition of G15, a selective GPER inhibitor, was able to prevent 27HC-stimulated cell growth in all three cell lines (Figure 2C and Figure S3A,B). Collectively, these data demonstrate that 27HC binds to GPER to induce the growth of ER− BC cells.

ERK1/2 are part of the GPR30 downstream pathway [37]. In response to 27HC we observed a dose-dependent increase in ERK1/2 phosphorylation (Figure 2D and Figure S3C,D), which was prevented by G15 (Figure 2E and Figure S3E,F).

**Figure 3:**
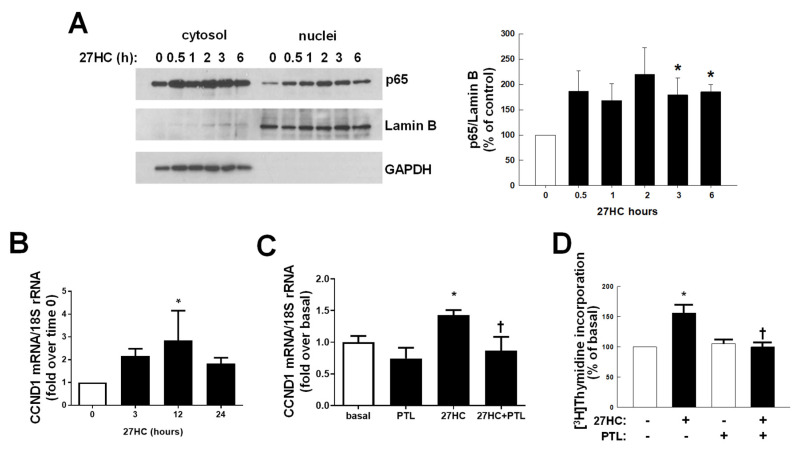
NFκB is part of 27HC-activated pathway and is required for cyclin D1 transcription. (**A**)**.** MDA-MB-231 cells were treated with 27HC (10^−6^ M) for 0–6 h, cytosolic and nuclear fractions were obtained, and Western blotting was performed for p65, Lamin B and GAPDH. Left panels display representative Western blots, and right panels provide summary data. Values are mean ± SEM, n = 5, * *p* < 0.05 vs. control (0 h). (**B**,**C**) QPCR analysis of cyclin D1 in MDA-MB-231 cells treated for increasing times with 27HC (10^−6^M) alone (**B**) or combined with parthenolide (PTL) (5 × 10^−6^ M) (**C**). (**D**) Cells were treated for 48h with 27HC (10^−6^ M) and PTL (5 × 10^−6^ M) alone and combined, proliferation was evaluated by ^3^H-thymidine incorporation over 6 h. * *p* < 0.05 vs. basal (untreated cells); † *p* < 0.05 vs. 27HC treated cells.

Furthermore, the nuclear factor κB (NFκB), a regulator of cell cycle [38], is also part of the GPER pathway [39] and we wanted to investigate its activation in the response to 27HC in our cell models. Cells were treated for increasing times (0.5 to 6 h) with the oxysterol to evaluate changes in nuclear localization of p65, an NFκB component (Figure 3A and Figure S4A,B). In a time-dependent manner, 27HC can increase p65 nuclear translocation, influencing the expression of cyclin D1 (CCND1), a known NFκB target gene [40] (Figure 3B and Figure S4C,D). Importantly, the presence of parthenolide (PTL), an NFκB inhibitor, prevented 27HC-dependent CCND1 upregulation (Figure 3C and Figure S4E,F) and, consequently, cell growth (Figure 3D and Figure S4G,H).

**Figure 4:**
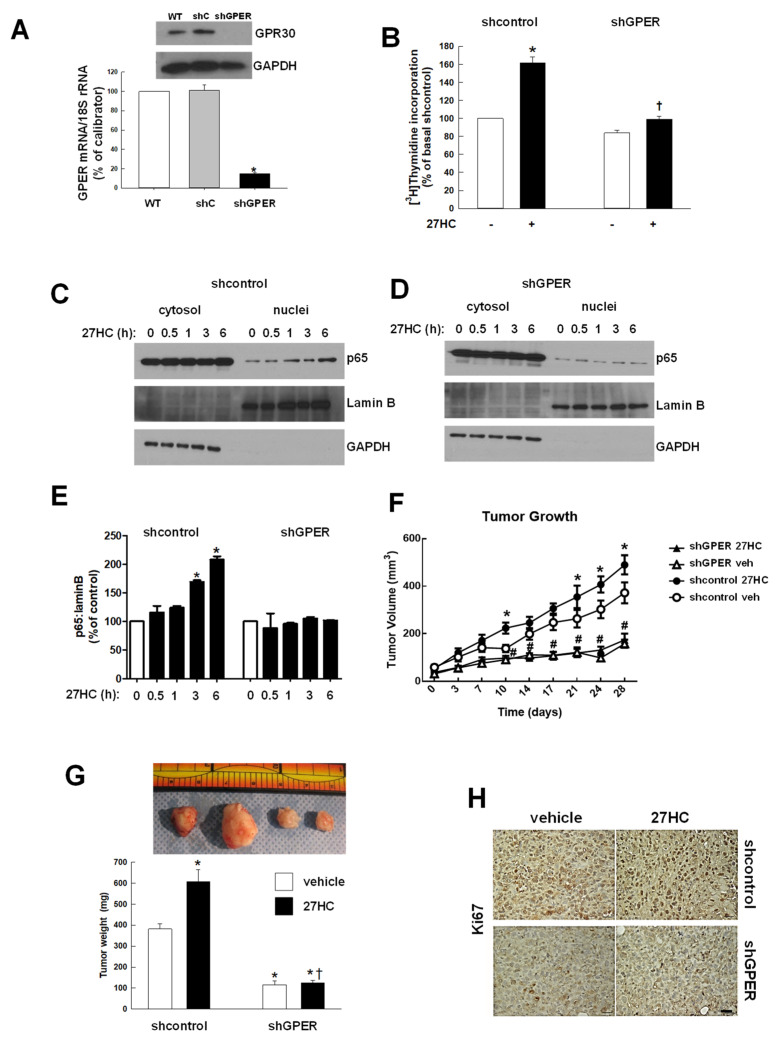
GPR30 mediates 27HC effects in ER− breast cancer cells. (**A**). GPER expression in parental (WT), shcontrol (shC) and stably GPER-silenced (shGPER) MDA-MB-231 cells were evaluated by Western blot analysis on whole-cell lysates (upper inset) and by real-time QPCR (lower graph). (**B**). Cells were left untreated (−) or treated (+) for 48h with 27HC (10^−6^ M) and proliferation was evaluated by ^3^H-thymidine incorporation. (**C**–**E**) ShC and shGPER MDA-MB-231 cells were treated with 27HC (10^−6^ M) for 0–6 h, cytosolic and nuclear fractions were obtained, and Western blotting was performed for p65, Lamin B and GAPDH. Representative immunoblots are shown in (**C**,**D**), and quantitative summary data are shown in (**E**). Values are mean ± SEM, n = 5, * *p* < 0.05 vs. control (0)**.** (**F**,**G**) Xenografts were initiated by implanting either shcontrol or shGPER MDA-MB-231 cells into the mammary fat pads of SCID mice. (**F**)**.** Growth curves of tumors were measured by caliper. * *p* < 0.05 vs. shcontrol vehicle, # *p* < 0.05 vs. shcontrol 27HC. (**G**) Representative tumors and final tumor weights. Values are mean ± SEM, n = 7, * *p* < 0.05 vs. shcontrol vehicle, † *p* < 0.05 vs. shcontrol 27HC. (**H**) Ki67 abundance was evaluated in tumors by immunostaining. Primary images were obtained with a 20× objective, scale bar 25 µm.

To further prove that GPR30 mediates 27HC-stimulated cell growth, GPER was stably silenced in MDA-MB-231 cells (shGPER) (Figure 4A). In these cells, 27HC no longer increased cell proliferation (Figure 4B). GPER-silenced MDA-MB-468 cells behaved similarly (Figure S5A,B). In shC cells, 27HC still has the ability to induce p65 nuclear translocation in a time-dependent manner, this effect is lost in shGPER cells (Figure 4C–E). Shcontrol and shGPER cells were grafted in immune-deficient mice, and while 27HC increased tumor volume and the mass of shcontrol tumors, it did not affect shGPER growth. Importantly, the growth rate of shGPER cells in vivo was slower than the shcontrol counterpart (Figure 4F,G). Staining for Ki67 confirmed that the proliferative index increased in response to 27HC in shcontrol cells but not in silenced cells. Additionally, vehicle-treated shGPER tumors showed less Ki67 stain than vehicle-treated shcontrol (Figure 4H).

### 3.3. 27HC Increases Cell Migration

**Figure 5:**
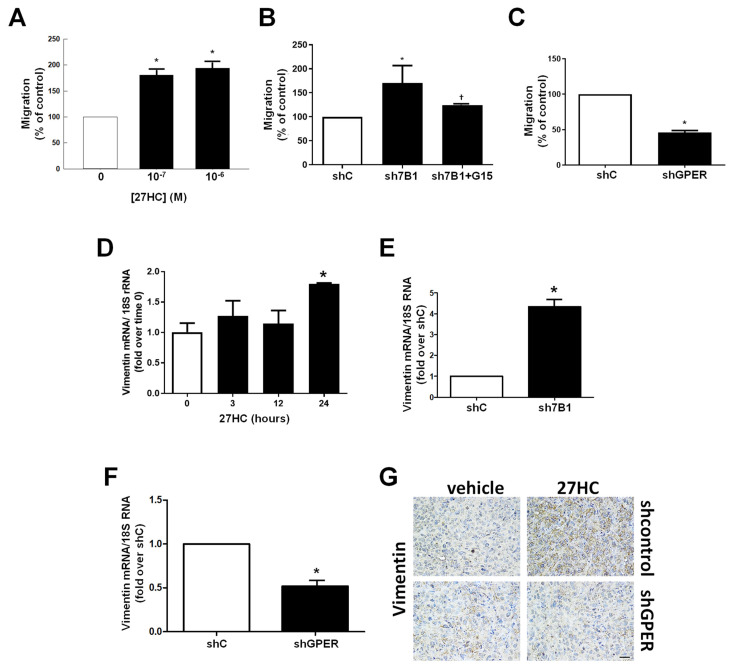
27HC via GPR30 induces EMT to sustain ER− BC cell migration. (**A**,**C**) Counts of MDA-MB-231 (**A**), shcontrol (shC) and shCYP7B1 (sh7B1) cells +/− G15 (10^−6^ M), (**B**) shC and shGPER cells (**C**) migrated through Boyden chambers. (**D**–**F**)**.** QPCR analysis of vimentin in MDA-MB-231 cells treated for the indicated times with 27HC (10^−6^ M) (**D**), shC and sh7B1 (**E**) shC and shGPER (**F**). (**G**) Vimentin abundance was evaluated by immunostaining in xenografts tumors of shcontrol or shGPER MDA-MB-231 cells. Images were obtained with a 20× objective, scale bar 25 µm. * *p* < 0.05 vs. basal (untreated cells) or shC cells; † *p* < 0.05 vs. sh7B1 cells.

We evaluated the effects of 27HC on cell migration and observed an increased motility in response to the oxysterol (Figure 5A and Figure S6A,B). Accordingly, shCYP7B1 cells displayed a more aggressive phenotype as evidenced by an increased migratory ability that was counteracted by the use of G15 (Figure 5B). By contrast, GPER gene silencing negatively impacted cell motility (Figure 5C and Figure S6C). Migration increases following the activation of epithelial-to-mesenchymal transition (EMT) genes, and we showed that 27HC modulates EMT markers in all three cell lines (Figure 5D and Figure S6D–H). Indeed, vimentin expression was increased in shCYP7B1 cells and decreased in shGPER cells (Figure 5E,F). Importantly, 27HC increased vimentin expression in xenograft tumors, as evidenced by a stronger IHC staining, which was not observed in samples from 27HC-treated shGPER xenografts (Figure 5G).

### 3.4. 27HC Requires GPER to Induce Tumor Angiogenesis

**Figure 6:**
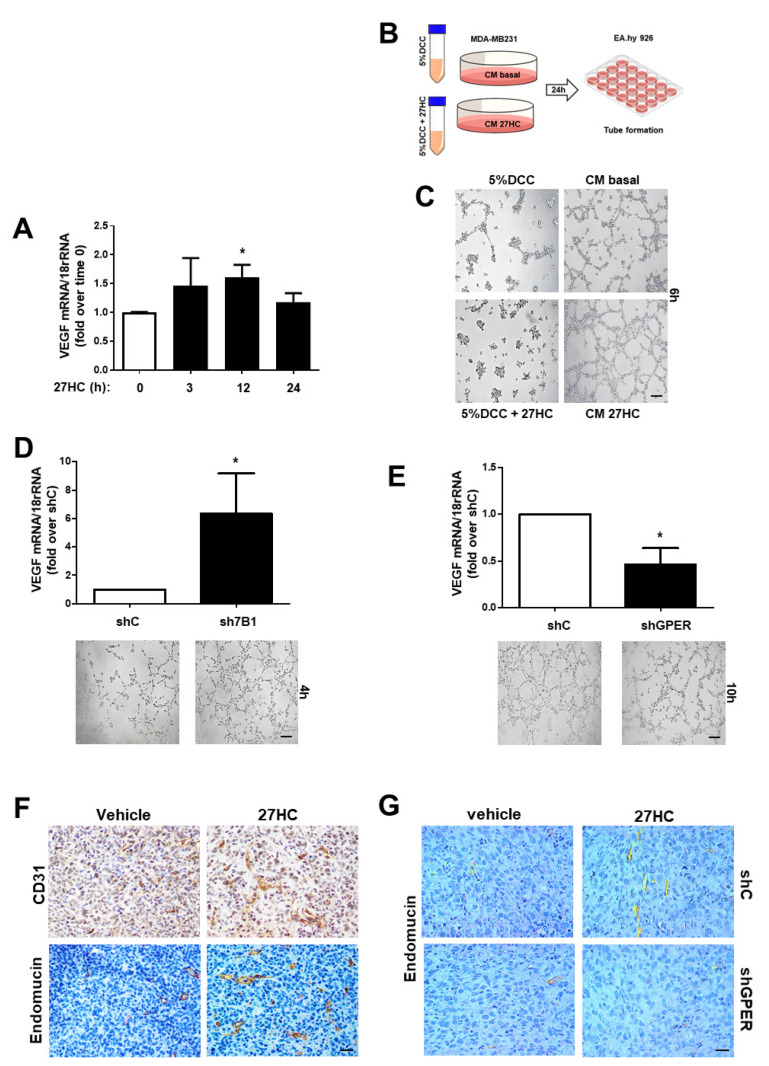
GPR30 is required to promote 27HC-induced angiogenesis in ER−BC cells. (**A**)**.** QPCR analysis of VEGF in MDA-MB-231 cells treated for the indicated times with 27HC (10^−6^ M) * *p* < 0.05 vs. basal (untreated) cells. (**B**) Graphical protocol used for the preparation of MDA-MB-231 conditioned medium (CM). (**C**–**E**) Tube formation in EA.hy926 exposed to conditioned media collected from MDA-MB-231 without (basal) and with 27HC (10^−6^ M) (**C**) and from shC, sh7B1 (**D**) and shGPER (**E**) * *p* < 0.05 vs. shC cells. Images were obtained with a 10× objective, scale bar 50 µm. (**F**,**G**). Abundance of CD31 and endomucin was evaluated by immunostaining in xenografts tumors of parental (**F**) or shC and shGPER (**G**) MDA-MB-231 cells. Images were obtained with a 20× objective, scale bar 25 µm.

GPER activation was linked to angiogenesis in BC cells [29]. Particularly, GPER signaling mediates VEGF gene transcription. The treatment of BC cells with 27HC evidenced a time-dependent increase in VEGF mRNA levels (Figure 6A and Figure S7A,B). The conditioned medium (CM) collected from 27HC-treated cells (Figure 6B) caused an increase in tube formation and branching (Figure 6C and Figure S7C,D). ShCYP7B1 and shGPER cells were used to further confirm that 27HC production by ER− BC cells activates GPER to support VEGF production and angiogenesis. VEGF expression is increased in shCYP7B1 compared to shcontrol cells, and endothelial cells exposed to CM from shCYP7B1 cells displayed more organized tube-like structures (Figure 6D). On the other hand, VEGF production is decreased in shGPER cells, and their CM was less effective in inducing tube formation in EA.hy926 cells (Figure 6E).

To establish the ability of 27HC to promote angiogenesis in vivo, xenografts from experiments indicated in Figure 1H,I and Figure 4F,G were evaluated by IHC for known markers of endothelial cells: CD31 and endomucin [41]. The expression of these markers increased in the presence of 27HC in xenografts from parental (Figure 6F) and shcontrol (Figure 6G) MDA-MD-231 cells but not in those from shGPER cells (Figure 6G). Vascular density was also decreased in shGPER compared to shcontrol xenografts (Figure 6G).

## 4. Discussion

Dysregulation of cholesterol homeostasis was associated with multiple types of cancer. Numerous studies showed increased levels of cholesterol in tumors as compared to normal tissues [42,43]. This is related to increased import via the low-density lipoprotein receptor (LDL receptor) and scavenger receptor class B type I (SR-B1, HDL receptor); inhibition of its export by the ABC transporters, ABCA1 and ABCG1; or deregulated cholesterol biosynthesis through the activation of sterol regulatory element-binding protein (SREBP); and the consequent upregulation of 3-hydroxy-3-methyl-glutaryl-coenzyme A reductase (HMGCR). Cholesterol increases the proliferation and motility of ER− BC cells [44,45]. Statins, which inhibit HMGCR, are more effective for preventing the growth of ER− than ER+ breast cancer cell lines [46,47]; additionally, they reduce TNBC cell metastases [48]. A recent study demonstrated that the moderate/strong expression of HMGCR is associated with prognostically adverse tumor characteristics such as higher histological grade, high Ki67, and ER negativity [49].

In recent years, increasing research interests have focused on the role of cholesterol metabolites oxysterols in tumorigenesis and cancer progression, with special attention toward 27HC, the most abundant circulating oxysterol in humans [8]. Since CYP7B1 is less expressed in TNBC tissues [19], in this study, we asked if cholesterol-related effects could depend on its conversion into 27HC. We proved this hypothesis by showing that: 1. TNBC cells silenced for CYP7B1 expression, manifest an increased proliferation when compared to control cells; 2. this increase is not observed when cells are grown in serum-deprived of lipoproteins. The impact of impaired 27HC catabolism on cell proliferation was previously shown for ER+ cells [14]. However, while 27HC acts through ERα in ER+ cells; here, we show that in ER− cells, the 27HC effect is related to GPER as demonstrated by [^3^H]E2 competition ligand-binding assay.

The finding of 27HC as a GPER ligand is not only relevant to breast cancer. Indeed, the role of GPER as a membrane-based estrogen receptor is still controversial, and conflicting results regarding the GPER-mediated signaling events in response to estrogens continue to emerge [50]. The binding of 27HC to GPER could help find an explanation for many of these controversies. A dual ligand ability has already been demonstrated for other GPCRs, such as sphingosine 1-phosphate receptor 2 (S1PR2), binding both Sphingosine 1-phosphate (S1P) and conjugated bile acids [51]; cannabinoid receptor 1 (CB1), binding both anandamide and 2-arachidonoylglycerol; and GPR119, binding lysophosphatidylcholine, oleoylethanolamide and 2-oleoylglycerol, among others.

We excluded the possibility that LXRs mediate the proliferative effects of 27HC since LXR activation inhibits ER− tumor cell proliferation [21,35]. This inhibition seems related to a decrease in cholesterol content as a consequence of the increased transcription of cholesterol efflux genes (ABCA1 and ABCG1), decreased LDLR expression (cholesterol uptake), and the accelerated degradation of HMGCR (cholesterol synthesis). The treatment of TNBC cells with 27HC instead causes an increase in proliferation, both in vitro and in animal models. Most importantly, this effect is not observed if GPER is stably silenced, further confirming the involvement of this receptor in 27HC-dependent proliferative effects. A study that evaluated the impact of a high-cholesterol diet (HCD) and 27HC on ER− breast tumor, using Met1 cells, a mouse ER− BC cell line, did not show any effect on the growth but found only a significant increase in metastasis formation [52]. However, a previous study, using MDA-MD-231 grafted in SCID mice fed an HCD, showed a significant increase in growth compared to xenografts grown in mice fed a control diet. Importantly, ezetimibe, a cholesterol-lowering drug, was able to prevent the effects of HCD [53]. Another study evaluated the effects of HCD on the xenografts growth of MDA-MD-231 cells in SCID mice. The authors not only demonstrated an increase in tumor size in mice fed for 10 weeks HCD, but also an increase in Ki67 positive cells [45]. 27HC levels parallel cholesterol, then, an increase in serum cholesterol is associated with increased 27HC, making the oxysterol a plausible effector for HCD. Additionally, the observation that 27HC does not increase Ki67 staining in xenografts from shGPER cells further supports the hypothesis of GPER involvement in mediating its action. The oncogenic role of GPER in TNBC is supported by the observation that Ki67 staining is clearly reduced in shGPER vehicle-treated xenografts, making this receptor a plausible target for BC therapy.

After binding to GPER, 27HC activates NFκB, which plays a prominent role in malignant proliferation, the prevention of apoptosis, resistance to chemotherapy, metastasis, and angiogenesis [54]. Studies from gene expression profiling analysis revealed that the NFκB pathway may be a key regulator of TNBC [55,56,57,58]. In our study, we show that 27HC increases p65 nuclear translocation in a GPER-dependent manner, up-regulating the transcription of its target gene, cyclin D1, a key proliferative gene.

The gene 27HC was also shown to be involved in the activation of EMT in MDA-MB-231, where it promoted STAT-3 phosphorylation, increased vimentin and MMP9 transcription, favoring a more aggressive phenotype [17]. Our data support these previous findings and define GPR30 as the receptor mediating 27HC-induced EMT and migration in ER− BC. The relevance of such an interaction is further proved by the behavior of stably silenced cells, where the higher 27HC levels (shCYP7B1) promote EMT and migration, while the absence of GPER (shGPER) causes a significant decrease in vimentin expression and, consequently, cell motility.

In both ER− and ER+ BC cells, 27HC is also involved in angiogenesis. Its angiogenetic effects occur following ROS production, STAT-3 phosphorylation, and HIF-1α transcription, and, ultimately, via VEGF synthesis [20]. Additionally, it was previously shown that, in SKBR3 cells, GPR30 activation by its synthetic ligand promotes the synthesis of factor(s) involved in angiogenesis. G1-treated SKBR3 cells promote tube formation in vitro and neoangiogenesis in vivo [29]. Our data link these independent observations, demonstrating that 27HC needs GPR30 to induce VEGF production and angiogenesis. This becomes clear from in vivo experiments comparing xenografts from MDA-MB-231 shcontrol and shGPER cells: 27HC increases endomucin staining only in control cells; the vehicle-treated group of shGPER xenografts shows less endomucin expression than the shcontrol counterpart.

## 5. Conclusions 

Collectively, our study demonstrates that 27HC is a new ligand for GPR30, and their signaling axis is involved in ER− BC progression, and particularly with neoangiogenesis. Interfering with the 27HC mechanism of action could represent a new targeted therapy for ER− BC, particularly for triple negative breast tumors, a type of cancer that still lacks specific interventions.

## Figures and Tables

**Table 1:** QPCR primers sequence.

Gene	Forward Primer (5′-3′)	Reverse Primer (5′-3′)
*ZEB1*	GCACCTGAAGAGGACCAGAG	TGCATCTGGTGTTCCATTTT
*VIMENTIN*	CCTTGAACGCAAAGTGGAATC	GACATGCTGTTCCTGAATCTGAG
*TWIST*	CGGGAGTCCGCAGTCTTA	TGAATCTTGCTCAGCTTGTC
*SNAI2*	CATGCCTGTCATACCACAAC	GGTGTCAGATGGAGGAGGG
*VEGF*	TGCAGATTATGCGGATCAAACC	TGCATTCACATTTGTTGTGCTGTAG
*CCND1*	CACGCGCAGACCTTCGT	ATGGAGGGCGGATTGGAA
*18S*	CGGCGACGACCCATTCGAAC	GAATCGAACCCTGATTCCCCGTC

## Data Availability

Data are available upon reasonable request.

## Supplementary Materials

The following supporting information can be downloaded at: https://www.mdpi.com/article/10.3390/cancers14061521/s1, Figure S1. 27HC increases ER− BC growth. Figure S2. 27HC activates proliferation of SKBR3 and MDA-MB-468 cells. Figure S3. GPR30 mediates 27HC effects in SKBR3 and MDA-MB-468 cells. Figure S4. NFκB/Cyclin D1 pathway is activated by 27HC in SKBR3 and MDA-MB-468 cells. Figure S5. GPR30 mediates 27HC-dependent proliferation in MDA-MB468 cells. Figure S6. Effects of 27HC on migration and EMT markers in SKBR3 and MDA-MB-468 cells. Figure S7. 27HC induces angiogenesis in ER− breast cancer cells.
