# Correction: Avena et al. 27-Hydroxycholesterol Binds GPER and Induces Progression of Estrogen Receptor-Negative Breast Cancer. *Cancers* 2022, *14*, 1521

**DOI:** 10.3390/cancers16233937

**Published:** 2024-11-25

**Authors:** Paola Avena, Ivan Casaburi, Lucia Zavaglia, Marta C. Nocito, Davide La Padula, Vittoria Rago, Jing Dong, Peter Thomas, Chieko Mineo, Rosa Sirianni, Philip W. Shaul

**Affiliations:** 1 Department of Pharmacy, Health and Nutritional Sciences, University of Calabria, Arcavacata di Rende, 87036 Cosenza, Italy; paola.avena@unical.it (P.A.); ivan.casaburi@unical.it (I.C.); luciazavaglia@hotmail.it (L.Z.); nocitomarta90@tiscali.it (M.C.N.);; 2 Marine Science Institute, University of Texas at Austin, Port Aransas, TX 78373, USA; 3 Center for Pulmonary and Vascular Biology, Department of Pediatrics, University of Texas Southwestern Medical Center, Dallas, TX 75390, USA; chieko.mineo@utsouthwestern.edu

## Error in Figure

In the original publication [1], there was a mistake in “Figure 6F” as published. In the images for CD31 staining for the vehicle-treated versus 27HC-treated tumors, we erroneously provided a region of the same microscopic field of view for a 27-HC-treated tumor. The error has been fixed by replacing the old incorrect image for the CD31 staining of a vehicle-treated tumor with the correct image for the CD31 staining of a vehicle-treated tumor.

The corrected Figure 6 appears below. The authors apologize for any inconvenience caused and state that the scientific conclusions are unaffected. This correction was approved by the Academic Editor. The original publication has also been updated.

## Figures and Tables

**Figure 6:**
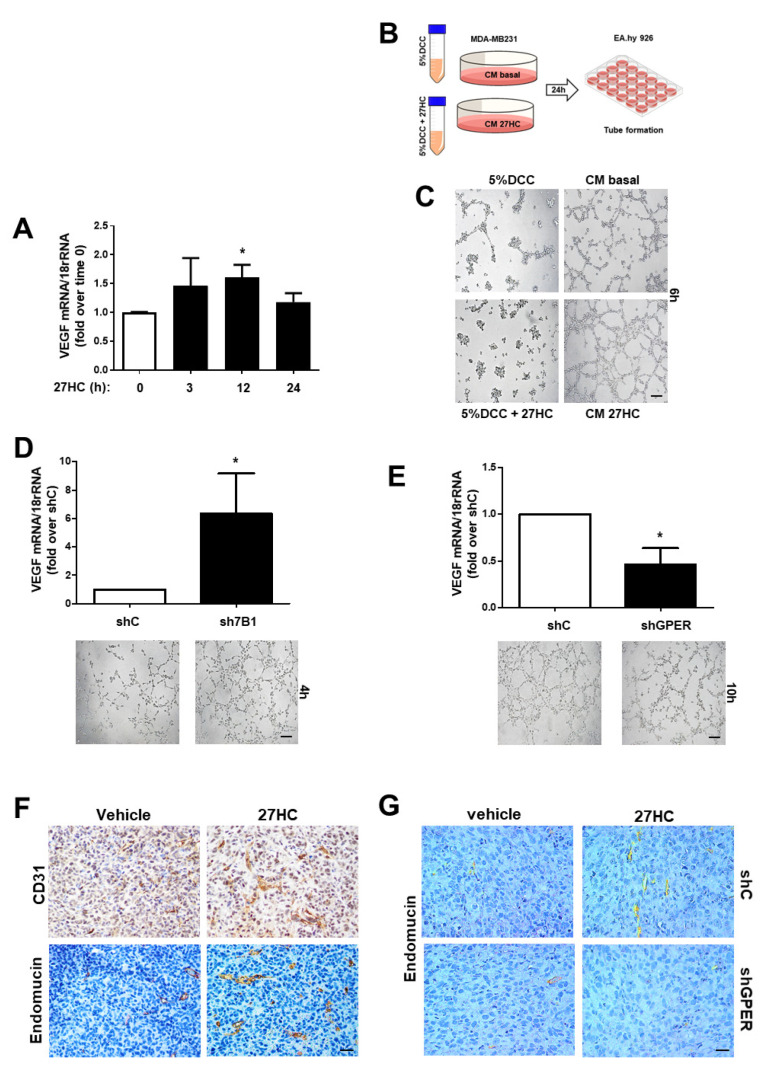
GPR30 is required to promote 27HC-induced angiogenesis in ER−BC cells. (**A**)**.** QPCR analysis of VEGF in MDA-MB-231 cells treated for the indicated times with 27HC (10^−6^ M) * *p* < 0.05 vs. basal (untreated) cells. (**B**) Graphical protocol used for the preparation of MDA-MB-231 conditioned medium (CM). (**C**–**E**) Tube formation in EA.hy926 exposed to conditioned media collected from MDA-MB-231 without (basal) and with 27HC (10^−6^ M) (**C**) and from shC, sh7B1 (**D**) and shGPER (**E**) * *p* < 0.05 vs. shC cells. Images were obtained with a 10× objective, scale bar 50 µm. (**F**,**G**). Abundance of CD31 and endomucin was evaluated by immunostaining in xenografts tumors of parental (**F**) or shC and shGPER (**G**) MDA-MB-231 cells. Images were obtained with a 20× objective, scale bar 25 µm.
