# Supplementary material for: 27-Hydroxycholesterol Binds GPER and Induces Progression of Estrogen Receptor-Negative Breast Cancer
Source: Cancers (Basel). 2022 Mar 16;14(6):1521. doi: 10.3390/cancers14061521 (PMC8946696; doi:10.3390/cancers14061521)
Supplement: Supplementary file 1 [file cancers-14-01521-s001.zip › cancers-1623343-supplementary.pdf]

Supplementary Data

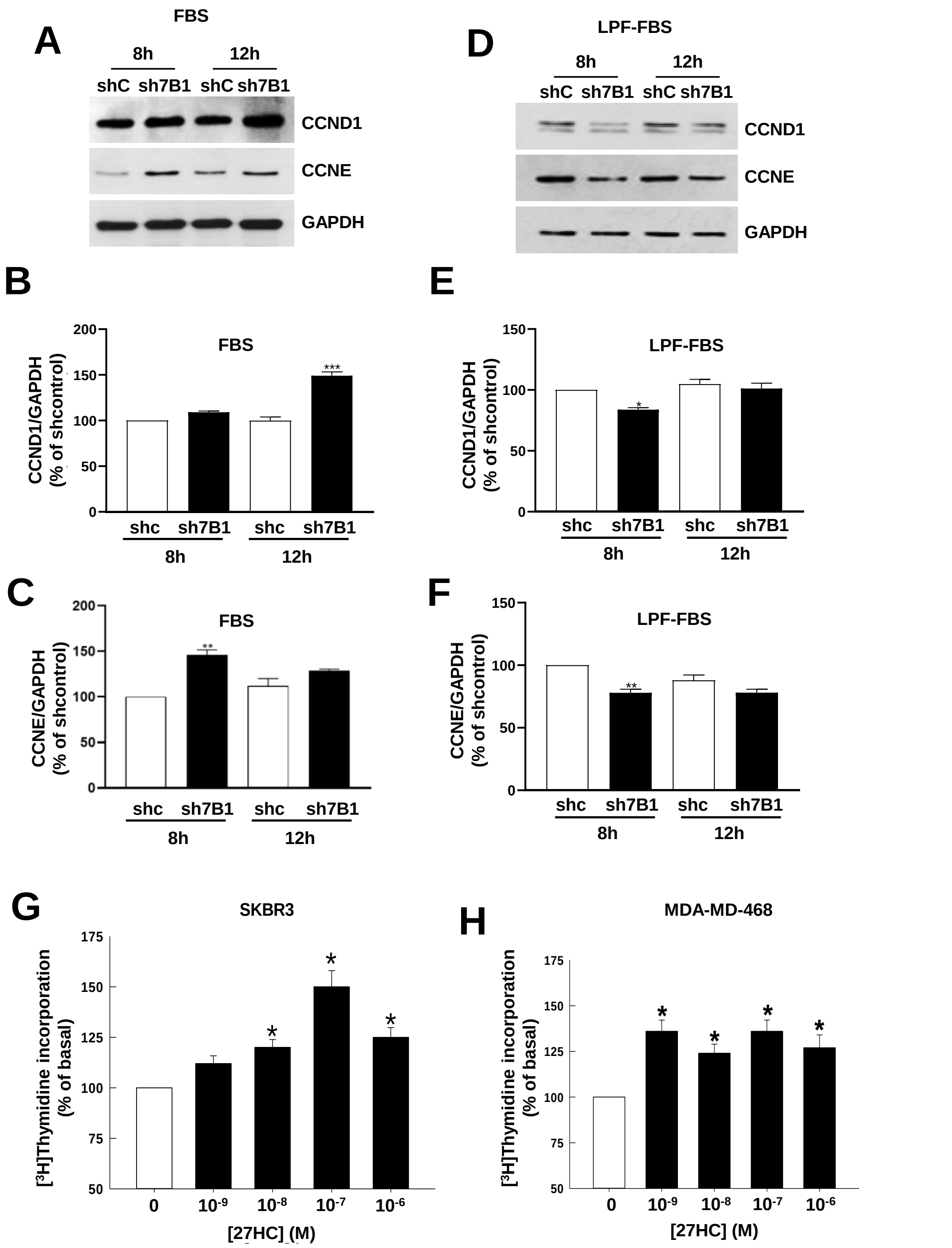

Figure S1. 27HC increases ER- BC growth **A-F.** Cyclin E and D1 expression was evaluated by SDS PAGE on shC and sh7B1 cells grown in the presence of 5% FBS (**A**) or 5% LPF-FBS **(D)** for the indicated times**.** Representative immunoblots are shown in **A and D** and quantitative summary data are shown in **B, C, E, F**. Values are mean±SEM, n= 3, *p<0.05 vs shC. **G, H.** Proliferation was evaluated by ^{3}H-thymidine incorporation in SKBR3 and MDA-MB-468 treated for 48h with increasing concentrations of 27HC, *p<0.05 vs 0 μM 27HC.

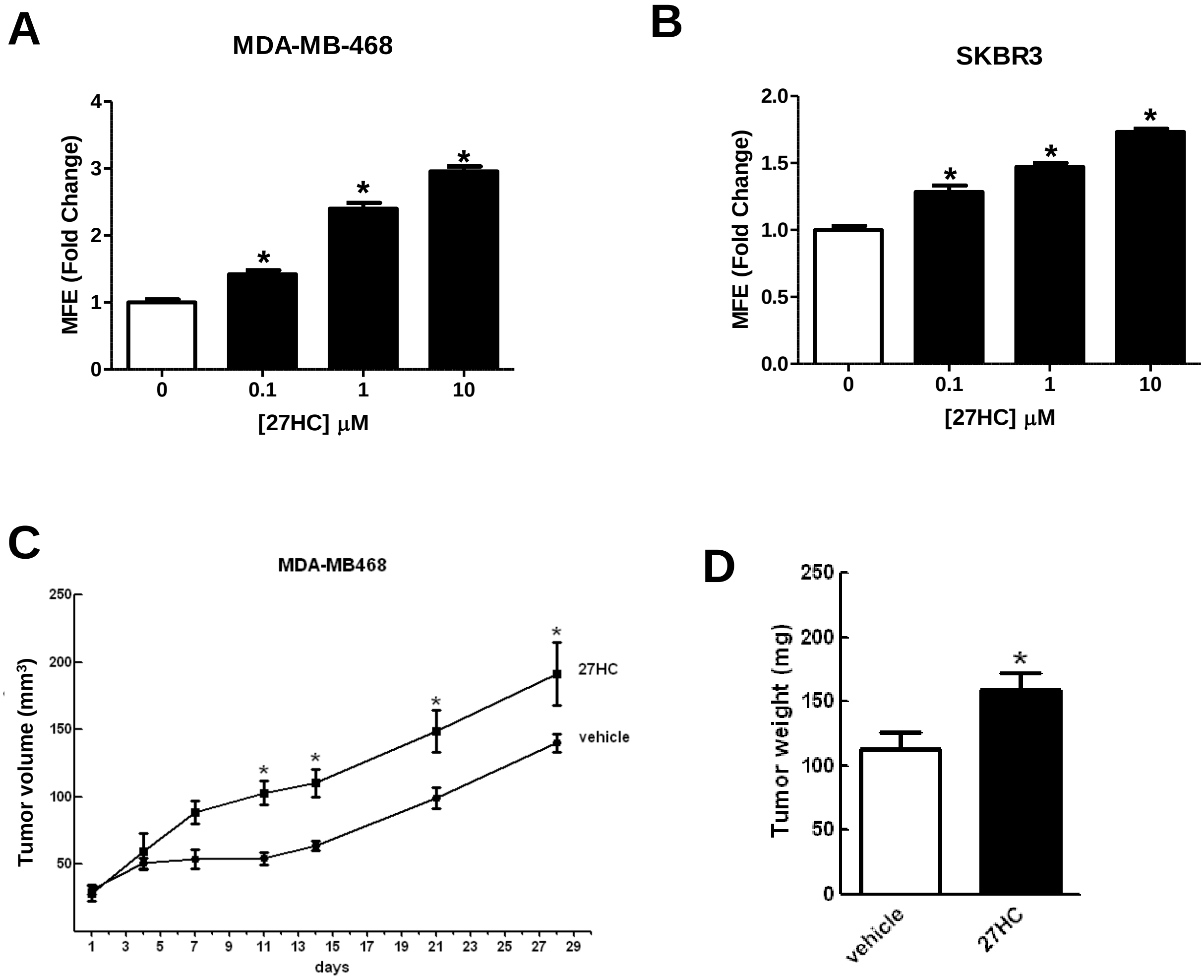

Figure S2. **A, B** 27HC activates proliferation of SKBR3 and MDA-MB-468 cells. MDA-MB-468 (A) and SKBR3 (B) cells were plated under non-adherent conditions and grown in the presence of 27HC (0-0.1-1-10 μM) for 5 days, and spheres > 50μm were quantified. Findings are expressed relative to mammosphere formation under basal conditions. Values are mean±SEM, n= 9, *p<0.05 vs basal. **C, D.** Xenografts were initiated using MDA-MB-468 cells, and the mice were administered vehicle versus 27HC for 28 days. **C.** Growth curves of tumors measured by caliper. **D.** Final tumor weights. Values are mean±SEM, n= 7, *p<0.05 vs vehicle.

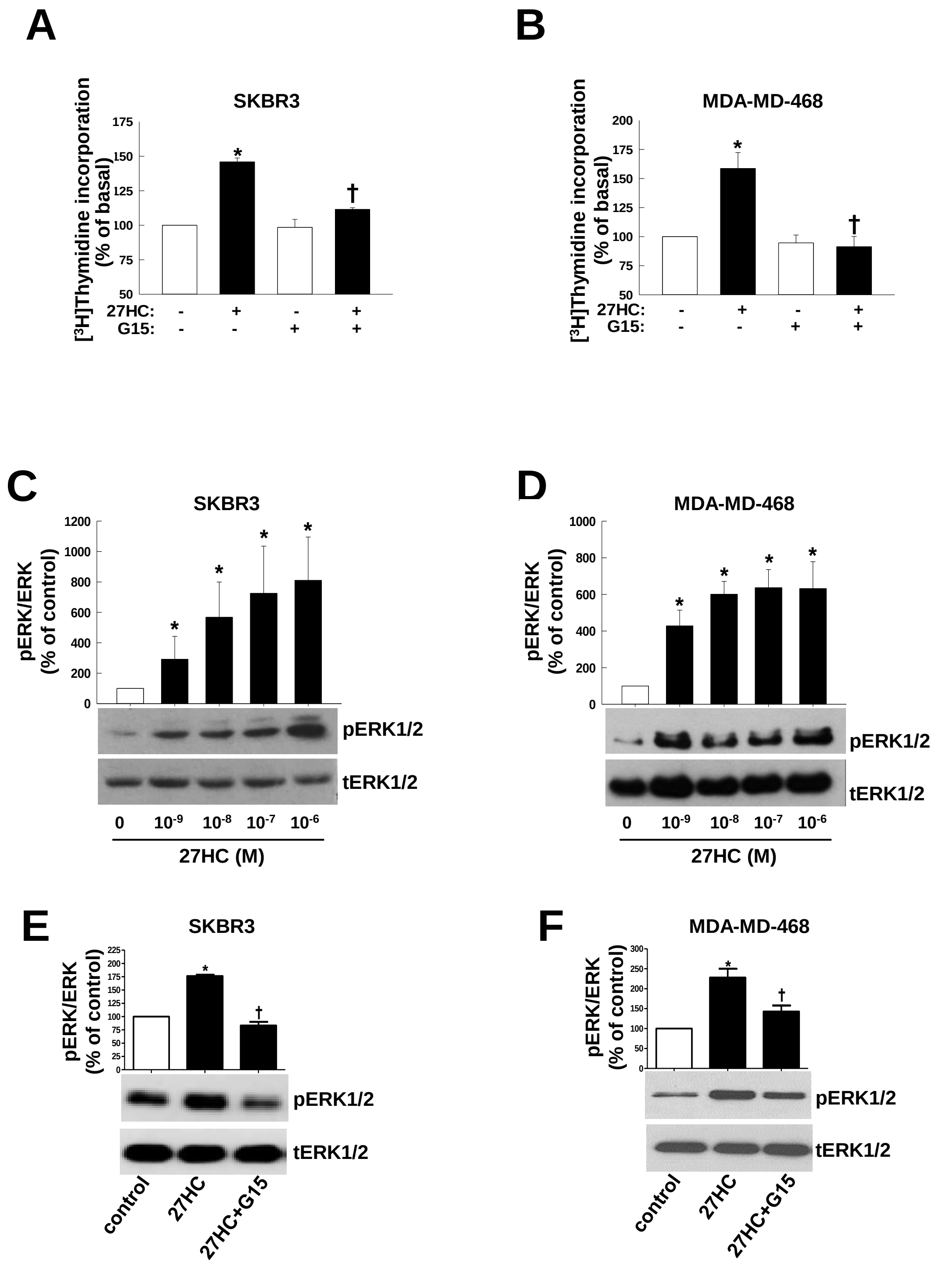

Figure S3. 27HC activates proliferation of SKBR3 and MDA-MB-468 cells. **A, B** ^{3}H-Thymidine incorporation was evaluated in SKBR3 and MDA-MB-468 cells treated for 48h with 27HC 10^{-6} M with or without G15 (10^{-6} M), *$p<0.05$ vs basal (untreated cells) †$p<0.05$ vs 27HC. **C-F.** Western blot analysis was performed to detect pERK1/2 or total ERK1/2 (tERK) on whole cell lysates from cells treated for 15 min with 0 to 10^{-6} M 27HC (**C, D**) or with 27HC 10^{-6} M and G15 10^{-6} M alone or in combination **(E, F)**. Graphs provide summary data, values are mean±SEM, n=3.

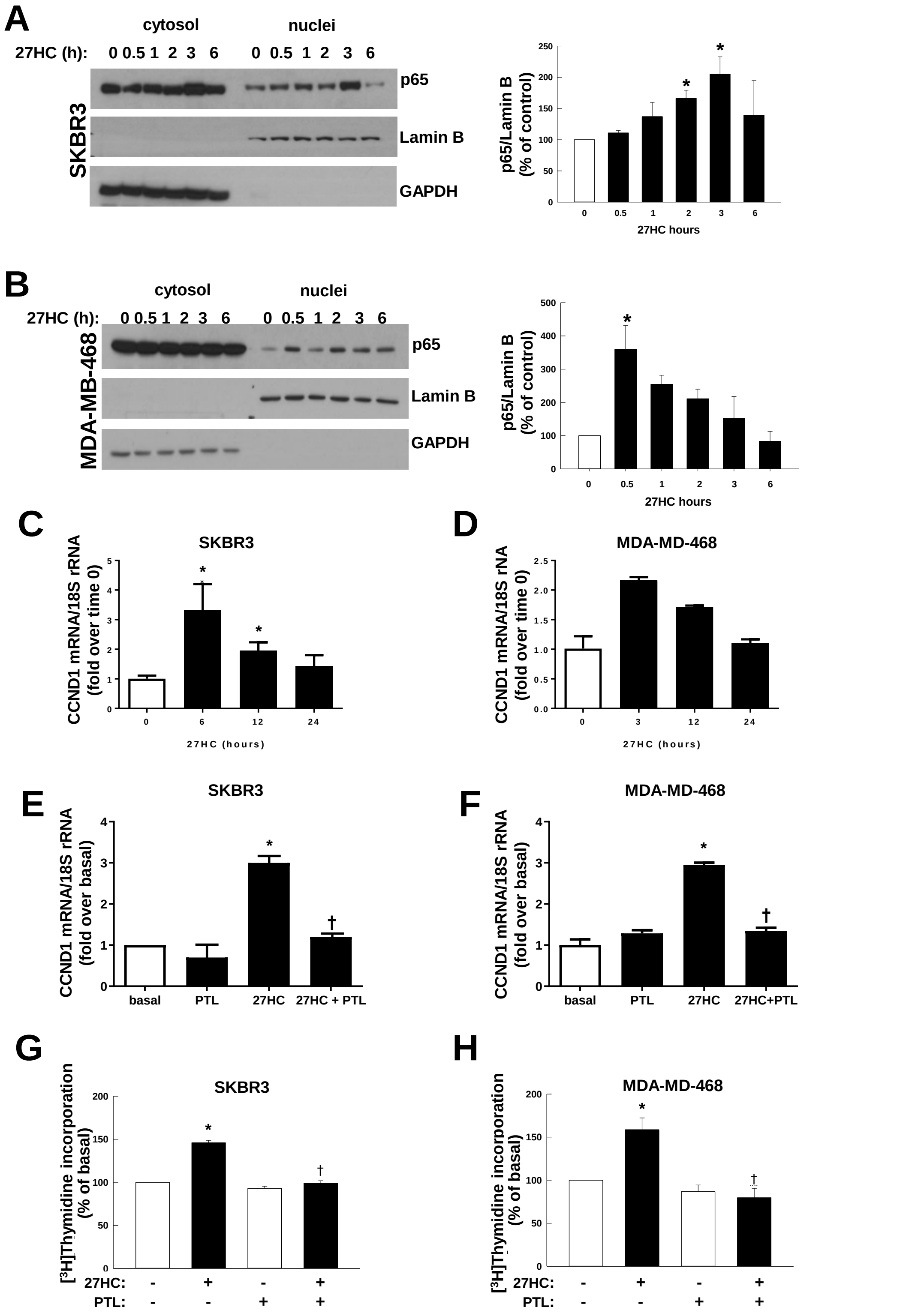

Figure S4. NFκB/Cyclin D1 pathway is activated by 27HC in SKBR3 and MDA-MB-468 cells. **A, B** SKBR3 and MDA-MB-468 cells were treated with 27HC (10^{-6} M) for 0-6 hours, cytosolic and nuclear fractions were obtained, and western blotting was performed for p65, Lamin B and GAPDH. Left panels display representative western blots, and right panels provide summary data. Values are mean±SEM, n= 3, *p<0.05 vs control (0 h). **C-F.** QPCR analysis of cyclin D1 in cells treated for increasing times with 27HC (10^{-6}M) alone (**C, D**) or combined with parthenolide (PTL) ($5x10^{-6}$ M) (**E, F**). **G, H.** Cells were treated for 48h with 27HC (10^{-6} M) and PTL ($5x10^{-6}$ M) alone and combined, proliferation was evaluated by ^{3}H-thymidine incorporation over 6 hours. *p<0.05 vs basal (untreated cells); †p<0.05 vs 27HC treated cells.

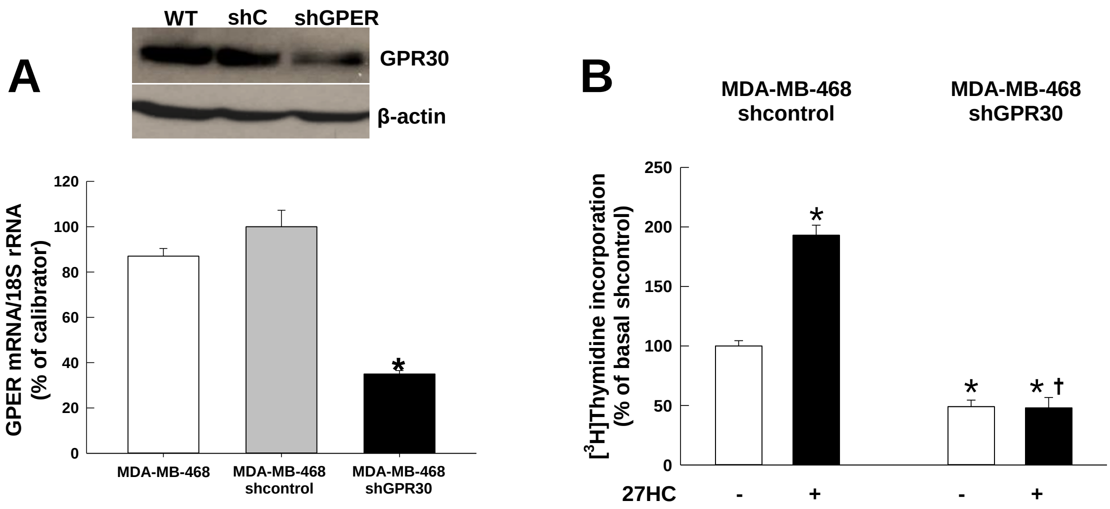

Figure S5. GPR30 mediates 27HC-dependent proliferation in MDA-MB468 cells **A.** GPER expression in parental (WT), shcontrol (shc) and stably GPER silenced (shGPER) MDA-MB-468 cells was evaluated by western blot analysis on whole cell lysates (upper inset) and by real-time QPCR (lower graph). **B.** Cells were left untreated (-) or treated (+) for 48h with 27HC (10^{-6} M) and proliferation was evaluated by ^{3}H-thymidine incorporation over 6 hours.

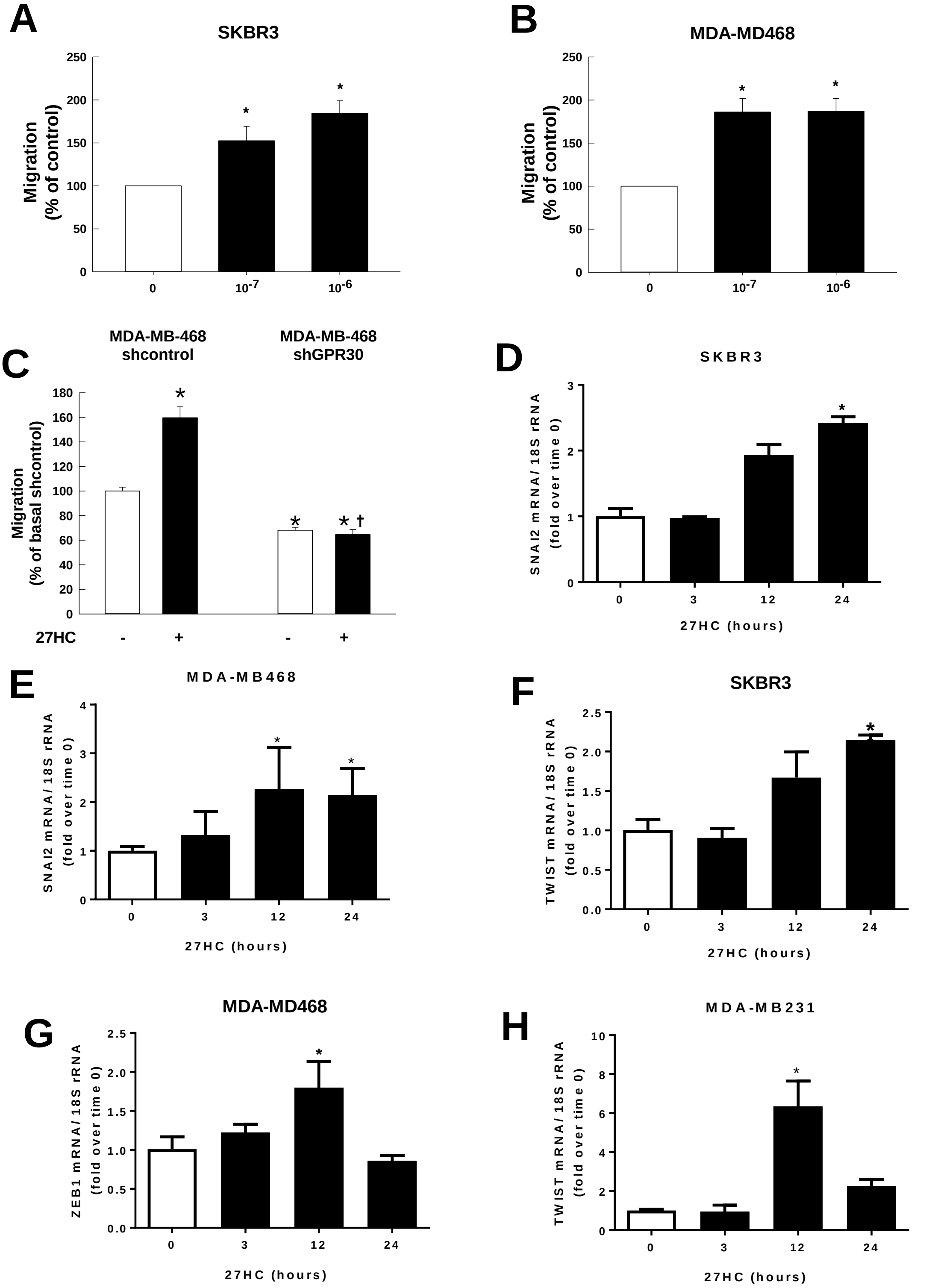

Figure S6. Effects of 27HC on migration and EMT markers in SKBR3 and MDA-MB-468 cells.. **A-C** Counts of **migrated** SKBR3 (**A**), MDA-MB-468 (**B**) cells treated with increasing doses of 27HC, MDA-MB-468 shC, and shGPER cells +/- 27HC **migrated** through Boyden chambers (**C**). **D-H** QPCR analysis of genes involved in EMT in SKBR3, MDA-MB-468, and MDA-MB-231 cells treated for increasing times with 27HC (10^{-6} M). *p<0.05 vs basal (untreated cells) or shC cells; +p<0.05 vs shC +27HC.

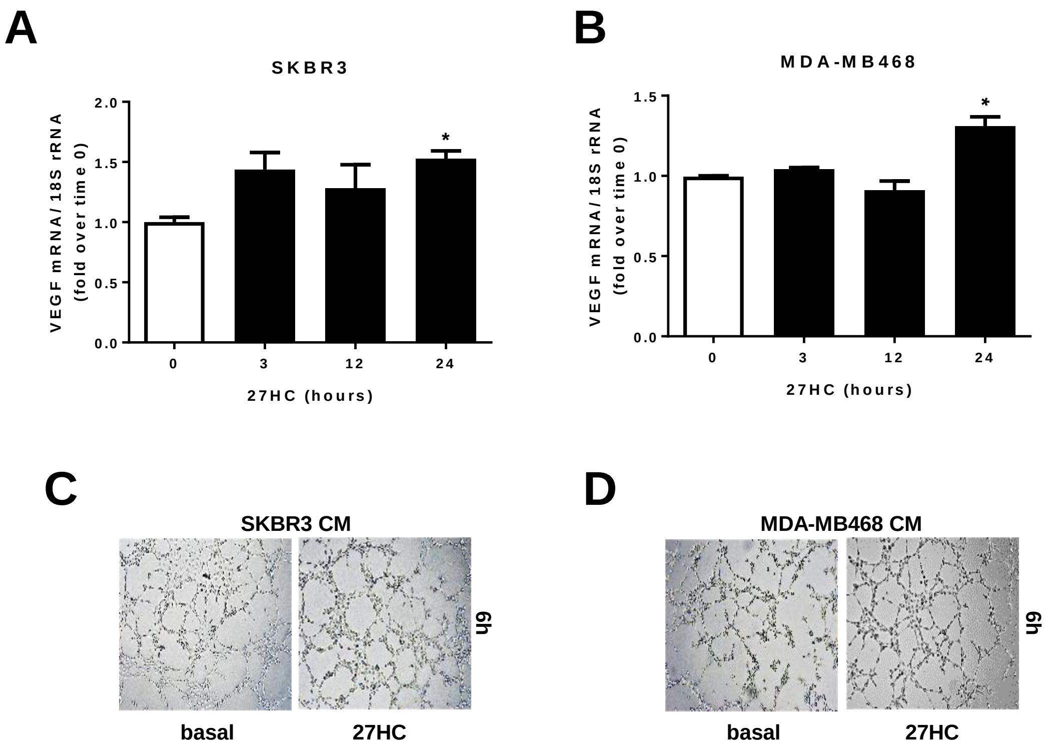

Figure S7. 27HC induces angiogenesis in ER- breast cancer cells. **A, B.** QPCR analysis of VEGF in SKBR3 (**A**) and MDA-MB-468 (**B**) cells treated for increasing times with 27HC (10^{-6} M). **C, D** Tube formation in EA.hy926 exposed for 6h to conditioned media collected from SKBR3 (**C**) and MDA-MB-468 (**D**) without (basal) and with 27HC. $p<0.05$ vs basal (untreated cells)
